# Context-dependent modulation of spatial attention: prioritizing behaviourally relevant stimuli

**DOI:** 10.1186/s41235-025-00612-x

**Published:** 2025-02-07

**Authors:** Noah Britt, Jackie Chau, Hong-jin Sun

**Affiliations:** McMaster University, 1280 Main Street West, Hamilton, ON Canada

**Keywords:** Context-specific behaviours, Altercentric cognition, Spatial cueing, Experience-driven attention, Driving, Scene grammar, Attentional learning

## Abstract

Human attention can be guided by semantic information conveyed by individual objects in the environment. Over time, we learn to allocate attention resources towards stimuli that are behaviourally relevant to ongoing action, leading to attention capture by meaningful peripheral stimuli. A common example includes, while driving, stimuli that imply a possibly hazardous scenario (e.g. a pedestrian about to cross the road) warrant attentional prioritization to ensure safe proceedings. In the current study, we report a novel phenomenon in which the guidance of attention is dependent on the stimuli appearing in a behaviourally relevant context. Using a driving simulator, we simulated a real-world driving task representing an overlearned behaviour for licensed drivers. While driving, participants underwent a peripheral cue-target paradigm where a roadside pedestrian avatar (target) appeared following a cylinder cue. Results revealed that, during simulated driving conditions, participants (all with driver’s licenses) showed greater attentional facilitation when pedestrians were oriented towards the road compared to away. This orientation-specific selectivity was not seen if the 3-D context was removed (Experiment 1) or the same visual scene was presented, but participants’ viewpoints remained stationary (Experiment 2), or an inanimate object served as a target during simulated driving (Experiment 3). This context-specific attention modulation likely reflects drivers’ expertise in automatically attending to behaviourally relevant information in a context-dependent manner.

## Introduction

Within the visual world, humans are constantly exposed to complex environments composed of a multitude of stimuli requiring varying levels of attention. Given our limited attentional resources, observers rapidly select which stimuli warrant attending. A recent surge in the literature has shown that the allocation of attention within dynamic visual scenes can be driven by factors such as behavioural relevance, scene semantics, or emotional significance (e.g. Beitner et al., 2021; Vater et al., 2022; Yu et al., 2023).

It has been well established that attention can be biased towards individual objects that convey inherent meaning. For example, a face with an angry expression attracts more attention compared to a face with a neutral expression (Okon-Singer et al., 2020). In addition, neutral, arbitrary objects without such inherent property that attract attention can become behaviourally relevant through learning in a specific context. An abstract experimental stimulus (e.g. a black square) that has been previously associated with a positive or negative outcome (e.g. monetary rewards, electric shock) can gain preferential attention in behavioural tasks when compared to objects that are not exposed to such association (Anderson et al., 2021; Failing & Theeuwes, 2018; Grégoire et al., 2021; Le Pelley & Newell, 2023; Schmidt et al., 2015).

Learning about the behavioural relevance of otherwise neutral objects can occur outside the laboratory, such as through repeated interactions with these objects during everyday behaviour in natural settings (Helbing et al., 2020; Kristjánsson & Draschkow, 2021). Thus, to better capture realistic stimulus-context learning, researchers have demonstrated that real-world stimuli could elicit attentional guidance patterns that relate to participants’ previous experiences. For example, researchers have presented participants with natural three-dimensional (3-D) scenes (e.g. a living room) and observed where participants fixated throughout the scene. Hayes and Henderson (2022) revealed that when freely viewing 3-D scenes, participants’ patterns of fixation were predominantly concentrated in locations of the scene that would be behaviourally relevant (e.g. a staircase) compared to less behaviourally relevant areas (e.g. empty corner of the room). This demonstrated that attention appears distributed towards spatial locations that are semantically meaningful within a specific visual context as a result of past exposures (also see Henderson et al., 2018; Hwang et al., 2011; Tachmatzidou & Vatakis, 2023; Wu et al., 2014; Yu et al., 2023), leading to attentional guidance via ‘scene grammar’ (Henderson, 2020; Võ et al., 2019; Wolfe et al., 2011).

In experimental settings with more specific task requirements, it has been demonstrated that the learned selectivity of attention could be context-dependent where patterns of attentional guidance are not generalizable to other, unrelated contexts (e.g. Brockmole & Võ, 2010; Castelhano & Heaven, 2010). Different from the aforementioned scene-based guidance in visual search studies, one of the most widely studied contextual manipulations is using action to induce motor-specific contexts. When anticipating an upcoming action, attention to features relevant to the action is facilitated within intermediate attention tasks. For example, Fagioli and Hommel (2007) demonstrated that while preparing for either a grasping or pointing action, concurrent perceptual task performance was facilitated for target features associated with either action: better target size processing before grasping action and better target location processing before pointing action (also see Han et al., 2020).

In Fagioli and Hommel (2007), the action context is provided through overt behaviours (grasping and reaching) required in the experiment. It has also been shown that attention modulation can be achieved by visual stimuli implying motor action despite the probability of the actual motor act being very low. This is due to internal models of behaviour being intrinsically activated when observing implied actions (e.g. seeing a posture indicative of a movement), oftentimes biasing attention towards locations or features that we expect to be related to the outcome of their implied action (Bach & Schenke, 2017; Bach et al., 2014). As an example, explicitly threatening body postures have been shown to reflexively guide attention as participants likely anticipate the outcome of certain postures (e.g. a kick to the left) despite never observing any action movements (Azarian et al., 2016; Bannerman et al., 2010; Wang et al., 2018). These results highlight that the accumulation of experiences with explicit actions—regardless of the experimental context or the commitment/observation of any actions—can result in attentional guidance due to implicit behavioural ramifications.

In the studies described above, the modulation of attention was triggered by individual action-related stimuli alone. Instead, we intended to explore whether attention could be modulated by a broader task context, while the target by itself does not offer motor implications without such context. In other words, the implied action is only present with a *combination* of the certain behavioural context and key stimulus feature that would otherwise not imply action.

We chose driving as the testing context as driving is an overlearned behaviour, and experienced drivers would likely develop an ‘attention set’ that would optimize the preparation for probable motor response in the event of a hazard (Crundall et al., 2012). In particular, this well-developed attention set would be activated automatically while driving, even when simulated. Previous research has shown that drivers are well-equipped to perceive hazards that appear away from their point of gaze (Crundall et al., 2002; Song et al., 2024; Wolfe et al., 2017). This indicates participants can use information throughout their peripheral visual field to inform their attention distribution despite not having to necessarily move their eyes (see Wolfe et al., 2020). In this case, drivers likely modulate their allocation of attention well before any overt motor response (e.g. change direction or speed of driving) even when the need for this motor action is currently very low but will likely be necessary soon after. We intended to exploit this attention set for licensed drivers by observing whether attentional guidance patterns while driving can differentially be distributed due to behaviourally relevant stimulus-context associations.

We focused on the processing of peripheral stimuli while requiring participants to maintain fixation to a centrally located stimulus. One concept to describe the spatial distribution of attention in the fronto-parallel plane is the useful field of view (UFOV). The UFOV measures the extent to which information can be extracted without eye or head movements from the visual field (Song et al., 2021; Wolfe et al., 2017) and performance in UFOV tasks has been shown to correlate with driving accident risk (Clay et al., 2005). Therefore, it is important to examine how attention affects peripheral processing in a driving context.

We used a modified spatial cueing paradigm where a peripheral spatial cue preceded the onset of the peripheral roadside target. In a typical version of this paradigm, participants respond to the onset of a target appearing in the peripheral visual field following the onset of a spatially uninformative cue (i.e. the cue is not predictive of the target location) in the same or a different location (with equal probability) as that of the target. In contrast to tasks which involve only one stimulus (target), spatial cueing tasks offer a more sensitive paradigm for measuring how attention shifts (often covertly) between locations (Chen & Wyble, 2018), specifically when orienting across stimuli in 3D space (see Britt & Sun, 2024; Haponenko et al., 2024). This way, the paradigm allows the researcher to investigate not only how attention arrives at the target location but also how attention shifts from original cue locations to the final target locations. For our task, a neutral cue (a cylinder) was presented before the target presentation, which was in the same or opposite hemifield.

In the current study, we conducted three experiments. Each experiment involved an experimental condition where licensed drivers drove in a driving simulator and moved forward along a straight road in a 3-D virtual reality environment. Participants were asked to follow and maintain their fixation on a lead vehicle. The target was a pedestrian-like avatar appearing roadside some distance ahead of the driver’s viewpoint. We measured drivers’ performance (reaction time) in discriminating a target feature (pedestrian’s arm position) that was not directly relevant to driving.

Roadside pedestrians represent a highly relevant peripheral stimulus that requires attention resources in the direction of where you expect them to be or where you expect them to go (Torralba et al., 2006). The most important (task-irrelevant) manipulation in the current study was the orientation of the pedestrian, which could be either facing towards the road (inward) or facing away from the road (outward). In the current experiment, the pedestrian never moved, but in the real world, with the driver moving along the road combined with the pedestrian’s inward orientation, in theory, there is a possibility of an impending collision between the vehicle and the pedestrian, should the pedestrian step out from the roadside. In contrast, the impending collision would be unlikely when the pedestrian assumed the outward orientation. Therefore, the performance difference when the orientation of the pedestrian was inward vs outward would be the indication of modulation of attention in a context-dependent manner. We anticipated that the inward orientation would garner more attention as a safety precaution due to it being perceived as an implicit hazard. To be specific, when comparing behavioural performance for the inward orientation to that for the outward orientation, the reaction time of target processing would be faster, and the cueing effect would show a greater extent of facilitation.

**Fig. 1:**
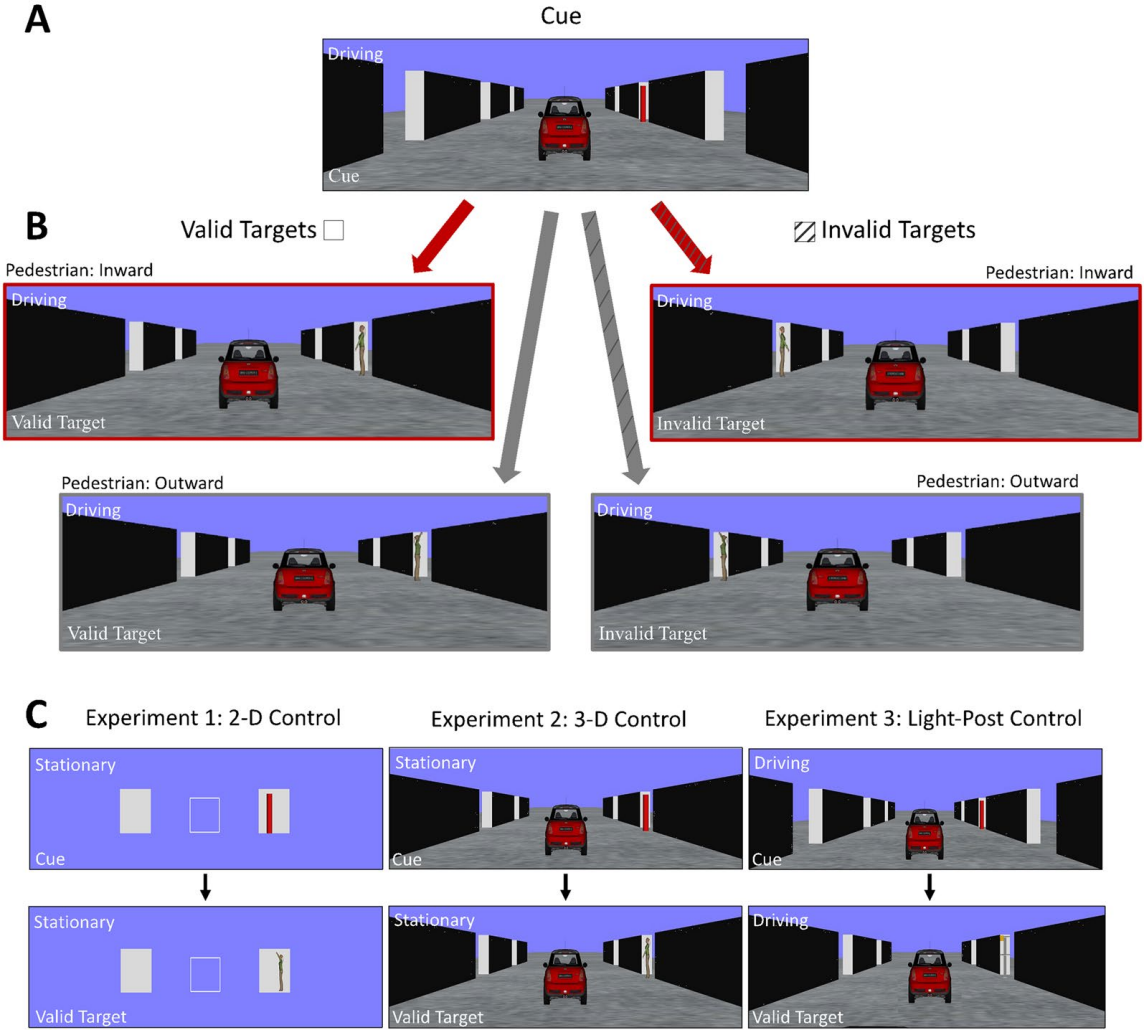
Example stimuli in the Experimental condition used in all three experiments. Illustrated is a **A** rightward cue stimulus followed by **B** valid targets (left) or invalid targets (right) with inward pedestrian orientations (top) and outward pedestrian orientations (bottom). Note that the pedestrian could appear invalidly or validly cued, oriented inward or outward, and with the arm positioned up or down, randomly on any trial. The coloured/patterned arrows are intended to match the results in Fig. 2. **C** Example stimuli for cue and target stimulus in the 2-D control condition of Experiment 1 (left), the 3-D control condition of Experiment 2 (middle), and the Light-Post control condition of Experiment 3 (right)

In addition to the experimental condition implemented in each of the three experiments in the current study, we also implemented three sets of control conditions where the target orientation was also varied between inward and outward orientations, but the behavioural context was removed through three different ways: 2-D stationary context with an isolated pedestrian target (Experiment 1), 3-D stationary context with a pedestrian target (Experiment 2), and driving with an inanimate light-post target (Experiment 3). See Fig. 1.

The control condition in Experiment 1 intended to isolate whether the difference in visual appearance of the target alone (without 3-D context) between inward and outward orientation would generate a difference in processing speed. The control condition in Experiment 2 intended to test whether attention modulation could still take place with the same 3-D information as that in the experimental condition, except that the motor act of driving is absent. The control condition in Experiment 3 intended to examine whether the forward self-motion alone would lead to attention bias by introducing an inanimate target object. In contrast to the experimental condition, as all the control conditions did not signal any possibility of impending collision, we anticipate that we would not see the performance difference seen in the experimental conditions between two orientations, either in RT or cueing effect.

## Methods

### Participants

All participants in each experiment reported normal or corrected-to-normal visual acuity and colour vision and were right-handed. All participants reported having a valid driver’s license and were naïve to the experiment. Participants first completed an informed consent form before participating. The study was approved by the McMaster Research Ethics Board, and participants were students in an Introductory Psychology course at McMaster University and rewarded with course credit for their participation. For each experiment, power analysis (Lakens & Caldwell, 2021) revealed a minimum n = 24 for 90% power to examine a cue validity × orientation × condition interaction in RT based on previous cueing effect magnitudes across depth (Britt et al., 2024). The power analysis did account for both within-subject and between-subject designs equally by ensuring the number of trials per condition was equal across all three experiments.

Experiments 1, 2, and 3 involved 28 participants (18–36 years of age, *M* = 19.6; 22 female and 6 male), 24 participants (18–21 years of age, *M* = 18.6; 18 female and 6 male), and 29 participants (17–28 years of age, *M* = 19.1; 19 female and 10 male), respectively.

### Stimuli and apparatus

The stimuli were back-projected onto a screen (207.4 cm tall × 144.8 cm wide, with a refresh rate of 60 Hz) positioned 150 cm in front of the participants. The entire setup was positioned inside a dark tent with minimal visual interference. The setup included a steering wheel device and gas/brake pedals that were positioned like in a regular car, and the participants sat at a comfortable distance from the device. The steering wheel was also equipped with response buttons.

This study presented depth information through monocular cues in a virtual 3-D environment. The 3-D virtual simulation was created using Vizard 7.0 virtual reality software. The measurement ‘vm’ was used to indicate metres within the virtual environment. As shown in Fig. 1, the virtual environment consisted of a ground plane and two sets of upright parallel walls (2.75 vm in height, 1 vm in thickness, 15 vm in length) oriented along the z-axis in the periphery. On both the left and right sides, there were multiple wall pieces with gaps of 6 vm between adjacent pieces. These wall pieces acted as left and right boundaries of a road on the ground, and the cue and target objects appeared in the gaps.

Across all three experiments, the identical experimental condition was implemented. Participants in the experimental condition were asked to use gas and brake pedals to undergo simulated driving (forward self-motion along the ‘road’) to follow a red lead vehicle (which also served as a point of fixation) and maintain a constant following distance (about 20 vm), making the visual angle for the lead red car to be 4.57° in width and 3.43° in height. The lead car had a constant speed of 10 m/s (36 km/h or 22 mph). To reduce the cognitive demand of the car-following task and, in the meantime, still create a sense of active control of the movement speed, the maximum driving speed of the participant’s car was set to be 10 m/s as well. As a result, participants could simply press hard on the gas pedal to maintain a constant following distance. Each trial started from a stationary state, and participants could accelerate with a maximum acceleration of 4.5 m/s^2^.

In addition to the car-following task, in a given trial, a red peripheral cylinder cue (2.66° tall, 0.57° wide) first appeared on the roadside (inside the gap between wall pieces) at a horizontal eccentricity of 8.29° when the participants’ viewpoint was 43 vm in the z-axis from the cue position. The pedestrian target (2.66° tall, 0.57° wide) then appeared at the same z-axis location as the cue and with an eccentricity of 9.09° when the participants’ viewpoint was 30 vm (in the z-axis) away. See Fig. 1A and B. The pedestrian avatar maintained a standing (not walking) posture and appeared with a green shirt and beige pants. Importantly, the pedestrian’s body was oriented either towards the road or away from the road and could either have their arm (farthest one from the driver) up in the air or down at their hip. Pedestrians were made to rotate around the y-axis 33° (inward) and 147° (outward), with 0° being facing perpendicular to the driving path. This way, both pedestrian orientations appeared completely perpendicular to the participant's line of sight at 100 ms after the target onset. This was intended to create a mirror image of the target for the two orientations. The same experimental condition was implemented in each of the three experiments and compared to three different control conditions implemented in the three experiments, respectively. All conditions, 2-D and 3-D, were tested in the same driving simulator setup.

For Experiment 1, the 2-D control condition included only the cue and target implemented in the experimental condition. All the context for the 3-D environment was removed. The red fixation vehicle in the experimental condition was replaced by a size-matched white box. In the periphery, one solid white box (width = 2.24°, height = 2.86°) appeared on either side of the fixation. The same cue and target as implemented in the experimental condition were presented with matching eccentricities within the peripheral white boxes.

In Experiment 2, the 3-D control condition included the central red fixation vehicle positioned 20 vm in front of the participant’s viewpoint. The participant’s viewpoint remained stationary at all times and was positioned 30 vm (along the z-axis) away from the cue and target locations. The peripheral cue in the shape of a red cylinder and peripheral pedestrian target appeared on either the left or right side of the display at identical horizontal eccentricities of 9.09°.

Lastly, in Experiment 3, the control condition was identical to the experimental condition except that the pedestrian target was replaced by an inanimate light-post target stimulus (2.66° tall, 0.71° wide). The light-post target contained two horizontal ‘arms’ positioned in the top and middle. A yellow square ‘light’ (0.28° tall, 0.28° wide) could appear on either arm at either the inward or outward positions. Overall, the four possible arm positions could match the configuration of the pedestrians’ arms. All three control conditions are illustrated in Fig. 1C.

### Procedure

Before beginning the experimental trials, participants completed an 18-trial practice block to become familiar with the environment and the response procedures. This practice block contained either a single condition (for Experiment 2 due to between-subject design) or both conditions (for Experiment 1 and Experiment 3 due to within-subject designs). Participants were given instructions verbally while seated at the steering wheel with the experimenter. For the experimental condition, participants began a trial by fixating on the central red vehicle. Participants were instructed to press the gas pedal with their right foot at the trial onset to follow and maintain a constant following distance from the lead red car vehicle. The timing of the stimuli was linked to the speed of the car, but ideal timings were as follows. After driving 10 vm, the cue appeared on either the left or right side for 100 ms. Following a random stimulus onset asynchrony (SOA) between 1000 and 1400 ms, the target appeared on either the left or right side for 500 ms. Participants were instructed to respond (as fast and as accurately as possible) to the pedestrian's arm position (or the light post’s light position in the control condition of Experiment 3) with an ‘up’ or ‘down’ button by pressing one of the two buttons with their right hand on the steering wheel. In all conditions, participants were instructed not to make eye movements to the best of their ability and to remain focused on the centre as indicated by the lead vehicle. Breaks were given between blocks of trials and did not have a fixed duration.

### Design

Each of the experimental and control conditions in all three experiments involved a factorial within-subject design of 2 (cue validity: valid vs invalid) × 2 (target orientation: inward vs outward) × 2 (arm position: up vs down). Targets were also equiprobable on the left and right side of the display. All levels of these variables are randomized in order in a block of 16 trials. The experimental and control conditions involved either within-subject (Exp 1 and 3) or between-subject (Exp 2) design.

Experiment 1 used a within-subject design where each participant took part in both the 2-D stationary control and experimental conditions. The experiment was split in half so that the first 13 blocks involved the 2-D control, while the last 13 involved the experimental condition, or vice versa. Participants underwent 416 total trials (208 per condition). The condition that appeared in the first and second half of the experiment was randomized across participants with an even number of participants starting with either condition.

Experiment 2 used a between-subjects design where each participant took part in either the 3-D stationary control or experimental condition. Participants underwent all 26 blocks (416 trials) in the same condition. The between-subject design was implemented in this experiment to ensure the strategy of attention allocation in the first condition would not contaminate responses in the second condition, as might happen with a within-subject design. After all, the visual appearance of the environment in the two conditions was very similar.

Experiment 3 used a within-subject design where both target types (Pedestrian, Light-Post) were blocked, and participants completed all trials with one type of target before the other. The number and order of trials/blocks/conditions were identical to that in Experiment 1.

### Data analysis

Analyses were carried out using RStudio (v2024.12.0) and the *afex* package (v1.4–1; Singmann et al., 2016). RT represents the delay between the target onset and the participant’s button press response on the steering wheel. In addition, we converted the RT measurements into their respective cueing effects. Cueing effects were calculated as the RT difference (per participant for each condition) between invalidly cued trials and validly cued trials (RT_Invalid_ – RT_Valid_). Positive cueing effects are indicative of a facilitation effect where RTs are shorter towards validly cued trials compared to invalidly cued trials.

Unplausible RTs longer than 1500 ms and shorter than 200 ms (2.05%, 3.25%, 2.96% for Experiments 1, 2, 3, respectively), responses before target onset (0.14%, 0.17%, 0.25%), trials with SOAs outside the expected range due to the removal of the participant’s foot from the gas pedal between cue offset and target onset (0.36%, 0.20%, 0.37%), as well as incorrect discrimination responses (3.02%, 1.56%, 1.53%), were excluded. Then, subsequent outlier data (3.75%, 3.93%, 3.85%) were also removed on either side of the RT distribution using a highly conservative threshold criterion of three times the mean absolute deviation of the median raw RT data per participant (Leys et al., 2013). Altogether, approximately 9.35%, 9.08%, and 8.96% of the trials were removed per experiment, respectively. Given error rates remained below 4% across all conditions, further accuracy analyses were not necessary. The following results were conducted only on the trials remaining following the exclusion criteria outlined in this section.

## Results

### Experiment 1: experimental vs 2-D stationary control condition

#### Reaction times

Mean RT values for each participant and condition were entered into a 2 (cue validity: valid vs invalid) × 2 (orientation: inward vs outward) × 2 (condition: experimental vs 2-D control) type III univariate three-way repeated-measures analysis of variance (ANOVA) model. The ANOVA revealed a significant main effect of cue validity (*F*(1,27) = 14.57, *p* < 0.001, η_p_^2^ = 0.35), a nonsignificant main effect of orientation (*F*(1,27) = 3.15, *p* = 0.087, η_p_^2^ = 0.10), and a significant main effect of condition (*F*(1,27) = 9.28, *p* = 0.005, η_p_^2^ = 0.26). In addition, significant interactions were found for cue validity × condition (*F*(1,27) = 13.22, *p* = 0.001, η_p_^2^ = 0.33) and most importantly for three-way cue validity × orientation × condition (*F*(1,27) = 4.68, *p* = 0.039, η_p_^2^ = 0.15). The cue validity × orientation (*F*(1,27) = 3.06, *p* = 0.091, η_p_^2^ = 0.10) interaction and orientation × condition (*F*(1,27) = 1.87, *p* = 0.183, η_p_^2^ = 0.07) interaction failed to reach significance^1^.

The main effect of cue validity revealed faster overall RT for validly cued trials (643.5 ms) compared to invalidly cued trials (668.2 ms). But to further investigate the cue validity × condition interaction, a simple main effect analysis was conducted on cue validity within either condition. For the 2-D control condition, a one-way repeated measures ANOVA revealed no simple main effect of cue validity (*F*(1,27) = 1.97, *p* = 0.172, η_p_^2^ = 0.07). However, the experimental condition revealed a significant simple main effect of cue validity (*F*(1,27) = 24.1, *p* < 0.001, η_p_^2^ = 0.47). These results indicate that while the 2-D control condition revealed no significant facilitation effects, the experimental condition demonstrated the significant facilitation effect. To explore the significant three-way interaction, we completed the following cueing effect analysis.

### Cueing effects

We explored the simple main effect of pedestrian orientation (inward, outward) on the cueing effects in separate one-way ANOVAs for each condition. The ANOVAs revealed a significant simple main effect of pedestrian orientation in the experimental condition (*F*(1,27) = 7.23, *p* = 0.012, η_p_^2^ = 0.21) but not in the 2-D control (*F*(1,27) = 0.11, *p* = 0.741, η_p_^2^ = 0.004). While driving, participants had significantly greater facilitation effects when the pedestrian was facing inward (50.5 ms) compared to outward (28.6 ms), but this effect was not present in the 2-D condition (inward: 7.7 ms, outward: 10.3 ms).

### Experiment 2: experimental vs 3-D stationary control condition

#### Reaction times

Mean RT values were entered into a 2 (cue validity: valid vs invalid) × 2 (orientation: inward, outward) × 2 (condition: experimental vs 3-D control conditions) type III three-way mixed ANOVA model. The ANOVA revealed a significant main effect of cue validity (*F*(1,22) = 22.05, *p* < 0.001, η_p_^2^ = 0.50), a significant main effect of orientation (*F*(1,22) = 17.93, *p* < 0.001, η_p_^2^ = 0.45), and a significant main effect of condition (*F*(1,22) = 39.43, *p* < 0.001, η_p_^2^ = 0.64). In addition, significant interactions were found for cue validity × orientation (*F*(1,22) = 8.57, *p* = 0.008, η_p_^2^ = 0.28) and most importantly for cue validity × orientation × condition (*F*(1,22) = 4.87, *p* = 0.038, η_p_^2^ = 0.18). The orientation × condition^2^ (*F*(1,22) = 1.39, *p* = 0.251, η_p_^2^ = 0.06) interaction and cue validity × condition (*F*(1,22) = 4.08, *p* = 0.056, η_p_^2^ = 0.16) interaction failed to reach significance.^2^

The main effect of the condition revealed significantly faster RT for the 3-D stationary control condition (627.4 ms) compared to the experimental condition (792.0 ms). In addition, the main effect of cue validity revealed significantly faster overall RT for validly cued trials (control: 615.4 ms, experimental: 763.8 ms) compared to invalidly cued trials (control: 639.5 ms, experimental: 820.8 ms). Also, the main effect of orientation revealed significantly faster RTs for pedestrian stimuli facing inward (i.e. towards the road; control: 618.4 ms, experimental: 786.5 ms) compared to facing outward (control: 636.5 ms, experimental: 797.6 ms). Note that Experiment 2 resulted in the fastest control condition and slowest experimental condition across the three experiments. This is likely a confound of the between-subjects design that was only introduced in Experiment 2 and was removed in Experiment 3.

#### Cueing effects

Similar to Experiment 1, using one-way ANOVAs, the analysis revealed a significant simple main effect of pedestrian orientation (inward vs outward) on the cueing effects in the experimental condition (*F*(1,11) = 8.77, *p* = 0.013, η_p_^2^ = 0.44) but not in the 3-D stationary control condition (*F*(1,11) = 0.52, *p* = 0.485, η_p_^2^ = 0.05). While driving, the participants had significantly greater facilitation effects when the pedestrian was facing inward (73.6 ms) compared to facing outward (45.6 ms) but not when stationary (inward: 25.7 ms, outward: 21.8 ms).

### Experiment 3: experimental vs light-post target control condition

#### Reaction times

Mean RT values were entered into a 2 (cue validity: valid vs invalid) × 2 (orientation: inward, outward) × 2 (condition: experimental vs ‘Light-Post’ control condition) type III univariate three-way repeated-measures ANOVA model. The ANOVA revealed all main effects and interactions to be significant: cue validity (*F*(1,28) = 12.47, *p* = 0.001, η_p_^2^ = 0.31), orientation (*F*(1,28) = 15.0, *p* < 0.001, η_p_^2^ = 0.35), condition (*F*(1,28) = 23.56, *p* < 0.001, η_p_^2^ = 0.46), cue validity × condition (*F*(1,28) = 9.06, *p* = 0.005, η_p_^2^ = 0.24), cue validity × orientation (*F*(1,28) = 9.50, *p* = 0.005, η_p_^2^ = 0.25), orientation × condition (*F*(1,28) = 21.34, *p* < 0.001, η_p_^2^ = 0.43), and most importantly cue validity × orientation × condition (*F*(1,28) = 9.99, *p* = 0.004, η_p_^2^ = 0.26).

The main effect of cue validity revealed faster overall RT for validly cued trials (656.0 ms) compared to invalidly cued trials (697.6 ms). Further simple main effect analysis was conducted to observe if both conditions elicited significant facilitation effects. One-way repeated measures ANOVAs revealed simple main effects of cue validity in both the Light-Post control (*F*(1,28) = 8.79, *p* = 0.006, η_p_^2^ = 0.24) and experimental conditions (*F*(1,28) = 14.60, *p* < 0.001, η_p_^2^ = 0.34)—consistent with facilitation effects in both conditions.

#### Cueing effects

As in Experiments 1 and 2, the cueing effect analysis revealed a significant simple main effect of pedestrian orientation (inward vs outward) in the experimental condition (*F*(1,28) = 18.04, *p* < 0.001, η_p_^2^ = 0.39) but not with the light-post target (*F*(1,28) = 0.04, *p* = 0.834, η_p_^2^ = 0.002). This again revealed that while driving, participants had significantly greater facilitation effects when the pedestrians were facing inward (64.1 ms) compared to outward (36.9 ms), but this effect was not found when the pedestrian was replaced by the light-post (inward: 32.0 ms, outward: 33.5 ms).

**Fig. 2:**
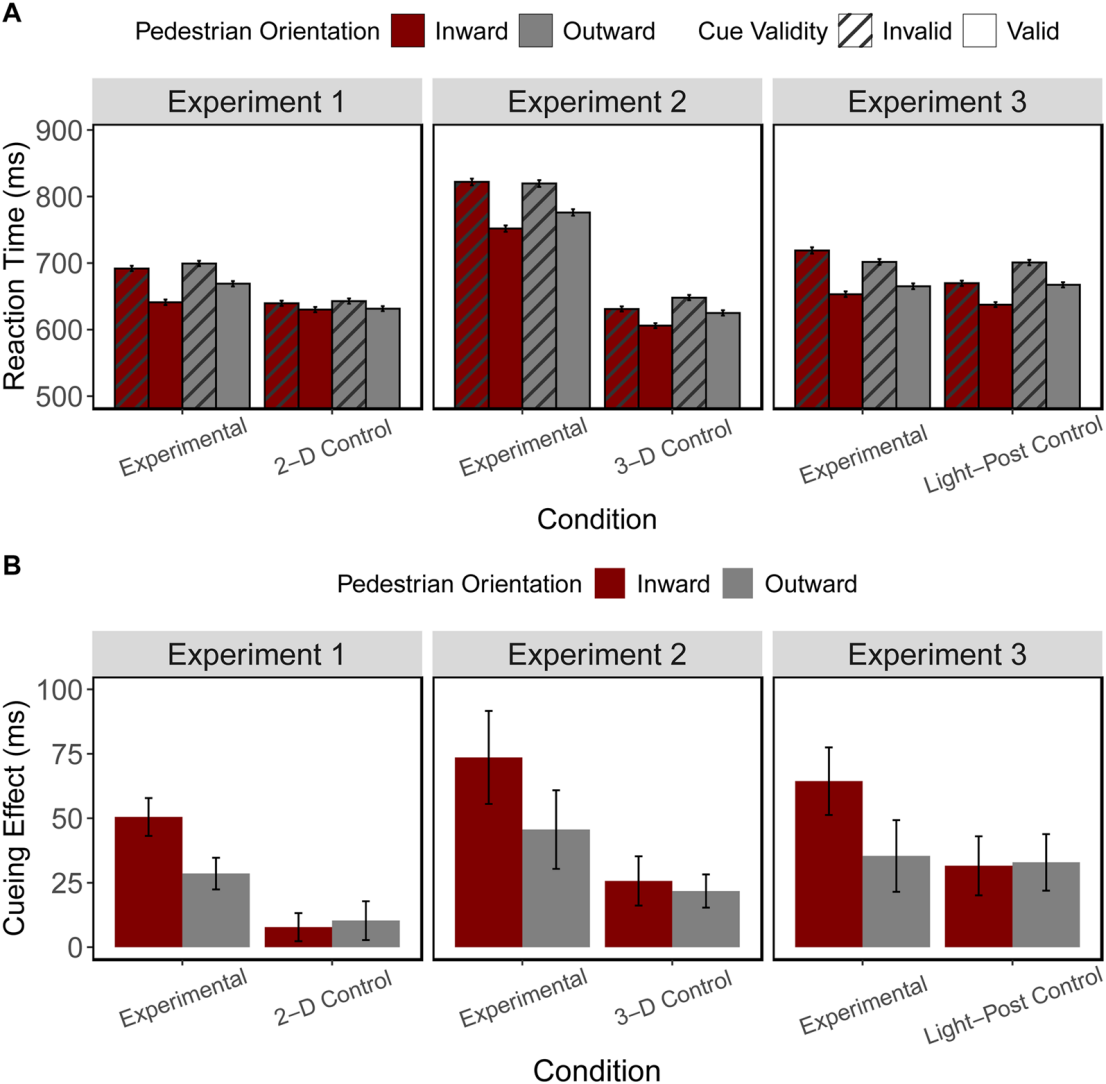
**A** Reaction Times data and **B** ‘Cueing Effects’ values in milliseconds for Experiments 1, 2, and 3. Cueing Effects used the formula RT_Invalid_ minus RT_Valid_. Error bars represent the standard error (Morey, 2008)

The descriptive statistics and comparisons of data between conditions for Experiments 1, 2, and 3 are shown in Table 1 and Fig. 2.

**Table 1:** Descriptive statistics for Experiments 1, 2, and 3

Orientation	Valid RT Invalid RT	Cueing effect	Error rate %	Valid RT Invalid RT	Cueing effect	Error rate %
Exp 1	Experimental Condition			2-D Stationary Control		
Inward	645.7 (4.0) 696.2 (4.1)	50.5 (7.3)	1.99 (0.26)	632.4 (4.0) 640.2 (3.8)	7.7 (5.4)	3.36 (0.40)
Outward	671.4 (3.9) 699.9 (4.1)	28.6 (6.1)	2.78 (0.34)	634.9 (3.7) 645.3 (3.9)	10.3 (7.5)	3.88 (0.46)
Exp 2	Experimental Condition			3-D Stationary Control		
Inward	752.2 (4.7) 825.8 (5.1)	73.6 (18.0)	2.16 (0.82)	606.4 (3.8) 632.1 (3.8)	25.7 (9.6)	0.68 (0.19)
Outward	776.3 (4.8) 822.0 (5.0)	45.6 (15.2)	2.52 (0.75)	626.3 (3.9) 648.1 (4.0)	21.8 (6.4)	0.84 (0.41)
Exp 3	Experimental Condition			Light-Post Target Control		
Inward	653.9 (4.3) 718.0 (4.7)	64.1 (13.1)	2.79 (0.37)	637.9 (3.7) 669.9 (3.9)	32.0 (11.5)	1.62 (0.21)
Outward	664.9 (4.2) 701.8 (4.3)	36.9 (13.9)	2.80 (0.38)	667.3 (3.8) 700.9 (4.0)	33.5 (10.9)	1.78 (0.24)

Reaction times and cueing effects (facilitation) are represented in milliseconds. Standard error values are corrected for within-subject variability (Morey, 2008)

## General discussion

Across all three experiments, our spatial cueing data aligned with our predictions: Cueing effects were significantly higher for pedestrians oriented inward (i.e. towards the road) compared to outward, but *only* in the experimental condition. This pattern of facilitation was not present in any of the three control conditions in the absence of the combination of context and target feature. Collectively, consistent differences in the cueing effects between the experimental and control conditions suggest that it was the contextual information that was able to evoke previously learned stimulus-context associations *only* when the context mimicked real-world driving conditions with roadside pedestrians.

In a driving situation, drivers are constantly exposed to many stimuli and being able to strategically allocate attention to driving-relevant events is very important. Attention modulation is the early process and perhaps always ongoing during driving. Overall, in the driving condition, expertise in modulating attention among visual events could be developed over years of driving experience, and eventually, the allocation process becomes automatic. The long-term learning of stimulus-context associations by licensed drivers that drove the selective attention focus on the roadside pedestrians with inward orientation during driving context reveals behaviour consistent with models of attentional learning (Hall & Rodrigues, 2019; Kruschke, 2011; Le Pelley et al., 2016). These models suggest, just like our results, that attention can be selectively tuned to be predominantly distributed according to prior experiences (i.e. spatial or feature priors) that can be context-sensitive (Bar, 2004; Fischer et al., 2020; Zhou & Geng, 2024).

Related to models of driving behaviour (Wolfe et al., 2020), driving experience allows for continued guidance of attention towards locations behaviourally relevant to the pedestrian solely based on the implied hazard associated with the prioritized target features. In this way, the concept of scene grammar (e.g. Henderson, 2020; Võ et al., 2019) applies to how drivers acquire information by the semantic value of specific target properties (i.e. the orientation of a roadside pedestrian) to benefit their safety. Accordingly, drivers can relatively rapidly ‘weight’ the stimulus dimensions that are most important to their ongoing behaviour in a given context. When stationary, we did not reveal any changes in cueing effects for inward and outward pedestrian stimuli, as the behavioural relevance of the stimuli did not necessitate any change in importance. However, when actively driving, the increased weighting of the inward pedestrian orientation was necessary because of the driver’s ability to expect potentially hazardous stimuli; this was not the case when driving but with the light-post target stimuli. Here, we emphasize the importance of contextual information in how drivers differentially allocate attention as a function of behavioural relevance and their safety. In other words, the term “context” here includes the entire behavioural relevance for dynamic attentional prioritization (rather than just the stimulus aspect).

To reiterate, the essence of our results is that the potential hazard of an inward-oriented pedestrian can bias attention when contextual information implies behavioural ramifications. Unlike the alternative (non-driving) literature showing planned actions (e.g. Fagioli & Hommel, 2007) or viewing action images (e.g. Schenke et al., 2016) can effectively bias attention towards action-congruent features or locations, the present results reveal a similar pattern, but only when both the stimulus and behavioural context are available. Here, based solely on the probabilities associated with learned characteristics of driving behaviour, the task-irrelevant orientation of the pedestrian target stimuli modulated attention as the experimental scenario suggested a potential roadside collision. Thus, we have furthered these theories of attentional learning by indicating a learned preparation effect based on conditional probability. Specifically, when inferring a potentially hazardous situation, previous memories from aversive experiences are easily accessible, prioritized over non-aversive memories, long-lasting, and can be retrieved by inherently neutral cues (Baczkowski et al., 2023; Bowen et al., 2018; Dunsmoor et al., 2015; Kalbe & Schwabe, 2022). We believe the combination of our inherently neutral pedestrian stimulus and the driving context rapidly selected memory representations to effectively guide attention to act in a predominantly defensive manner.

Furthermore, a secondary finding of this study that warrants explanation is that the inward orientation resulted in significantly faster RTs for all conditions involving a pedestrian except the 2-D control condition (showing an insignificant simple main effect of orientation). We believe this evidence supports an altercentric account of attention via our social pedestrian targets (Kampis & Southgate, 2020). Altercentric attention would predict that the orientation of nearby social agents modulates the focus of attention of the observers. We believe the RT data accurately reflect this spatial relation of the participant and pedestrian, and the disappearance of this effect of orientation only in the 2-D control condition would reflect the disappearance of depth information, thus removing the congruent alignment of the peripheral pedestrians’ orientation (i.e. facing inward towards the centre of the road) and participants’ central fixation (i.e. also facing towards the centre of the road). In this way, taking the perspective of the UFOV, the pedestrian targets appearing inward may appear to be more central than those oriented outward. The distribution of attention may then appear to be more focal in these circumstances due to the congruent alignment of attention, thereby improving the speeded response to target stimuli in the periphery.

Another interesting finding in our study is the difference in performance for pedestrian and light-post targets found in Experiment 3. It is reasonable to argue that the movable nature of the pedestrians led to the orientation effect. Beyond the context of attention modulation for the potential danger of impending collision, social perception of animacy has been well documented. There may be a priority in attentional processes for animate objects as they carry survival-relevant and social information. For example, it has been demonstrated there is a relation between attention bias towards animate over inanimate objects (shown through the dot-probe paradigm) and participants’ autistic traits (Yang et al., 2024). To confirm such interpretation in our paradigm, future research could test other animate target objects such as animals (e.g. roadside deer). In addition, the relation between driving experience (e.g. years after acquiring a driving license) and the attention bias towards pedestrians with inward orientation could be examined to reveal whether the attention bias in this situation is learned in a task-specific context. We speculate that such attention bias would be task-specific and go beyond sensitivity to animate objects in a general social context.

## Conclusion

Overall, the difference in cueing effect between the experimental and control conditions found in our study suggests that contextual information can evoke previously learned stimulus-context associations. The visual and behavioural context has the capacity to affect how participants prioritize their attention allocation towards behaviourally relevant stimuli. From an applied perspective, it is perhaps worthwhile to develop a battery of testing scenarios like the one in this study for attention allocation during driving, as this type of test could offer a more sensitive measure of driving expertise. This kind of testing would offer unique value that complements typical tests for drivers’ overt motor behaviours while driving.

## Significance statement

The present research highlights how our attention can be influenced by familiar real-world contexts. We found that drivers are more likely to focus on pedestrians oriented toward the road compared to those oriented away from the road, suggesting a learned defensive behaviour. This attentional bias only appeared in driving-like scenarios and the presence of pedestrians, indicating that our past experiences on the road shape how we pay attention to potential hazards. In real-world driving, such modulation of attention likely takes place well before motor planning and subsequent overt motor behaviours (such as easing on the gas and preparing to change lanes or brake) and can potentially offer a set of sensitive testing regimes for driving expertise. These insights are crucial for understanding how drivers perceive and respond to their environment, which can inform the driver training programs and design of safer road systems. Additionally, this research has direct ramifications for the fundamental research on how attention can be guided by behaviourally relevant cues.

## Data Availability

The datasets are available at OSF Repository. The data will be made public upon publication.

## Footnotes

1 Despite the insignificant orientation × condition interaction, we performed simple main effect analyses for the effect of pedestrian orientation in each condition and revealed a simple main effect of pedestrian orientation in the experimental condition (*F*(1,27) = 6.21, *p* = 0.019, η_p_^2^ = 0.19) but not in the 2-D control condition *F*(1,27) = 0.33, *p* = 0.571, η_p_^2^ = 0.01)—aligning with participants reacting quicker to inward-oriented pedestrians in the experimental condition only.

2 Despite the insignificant orientation × condition interaction, we again conducted one-way repeated measures ANOVAs to quantify the significant simple main effects of cue validity in both the 3-D stationary control (*F*(1,11) = 9.59, *p* = 0.010, η_p_^2^ = 0.47) and the experimental driving conditions (*F*(1,11) = 13.85, *p* = 0.003, η_p_^2^ = 0.56), revealing consistent facilitation effects in both conditions.

## Declarations

### Open practices statement

This study’s design and its analysis were not pre-registered.
